# Game Dynamics in Professional Padel: Shots Per Point, Point Pace and Technical Actions

**DOI:** 10.3390/sports12080218

**Published:** 2024-08-12

**Authors:** Iván Martín-Miguel, Bernardo Almonacid, Diego Muñoz, Bernardino Javier Sánchez-Alcaraz, Javier Courel-Ibáñez

**Affiliations:** 1 Department of Musical, Plastic and Corporal Expression, Faculty of Sport Sciences, University of Extremadura, 10003 Cáceres, Spain; ivanmartinmiguel97@gmail.com; 2 Department of Electrical Engineering, Higher Polytechnic School, University of Jaén, 23009 Jaen, Spain; balmona@ujaen.es; 3 Faculty of Sport Sciences, University of Murcia, 30100 Murcia, Spain; bjavier.sanchez@um.es; 4 Department of Physical Education and Sports, Faculty of Education and Sport Sciences, University of Granada, 52005 Melilla, Spain; courel@ugr.es

**Keywords:** performance, racquet sport, game analysis, fatigue, gender

## Abstract

This study aimed to determine the distributions of number of shots per point, point duration, point pace and technical actions during the match in professional men and women padel players. A total of 35,145 hits (3239 points; men = 1602 and women = 1637) were analyzed in 20 matches corresponding to quarterfinal, semifinal and final matches of the World Padel Tour 2020 season. The observations were conducted through systematic observation and involved a total of 32 players (16 men and 16 women). Men’s points lasted 13.5–14.8 s, comprising 10–11 hits, resulting in a ratio of 0.80 shots per second, with no differences between sets. Women’s points lasted longer than men’s (14.4 to 16.2 s, *p* = 0.011) but included a similar number of shots per point, resulting in a lower rhythm of play (0.73–0.75 shots per second, *p* < 0.001), particularly in the third set (*p* = 0.004). Volleys, lobs and directs were used in >60% of the points, with a frequency of one to three times per point. Bandejas were used in 50–60% of the points, with a frequency of one to two times per point. Backwalls and flat smashes were used at least once in 30–48% of the points. Selective actions had a greater frequency of use in the third set (i.e., flat smash and smash x3 in women; forehand/backhand volleys and side-wall forehand in men), suggesting occasional changes in the style of play, but likely explained by the onset of fatigue. In conclusion, game volumes, intensity and actions remained broadly similar throughout professional padel matches.

## 1. Introduction

Padel is becoming one of the most popular racket sports worldwide, with 25 million regular practitioners and a federative presence in over 90 countries [1]. Accordingly, the scientific interest in padel has seen exponential growth, particularly addressing performance-related topics to better understand players’ behaviors during the competition and aid coaches in the development of effective training programs simulating match-play conditions [2]. The current understanding of padel performance is limited to the game’s physical demands (volume and intensity) and the effectiveness of particular technical actions in the different areas of the court [3]; however, little is known about its relationship. Because performance relies on the ability to execute rapid and effective technical actions repeatedly during a match, volumes, intensity and the frequency of particular technical actions, which can be altered because of fatigue, a holistic understanding of padel game demands is needed.

Typically, a professional padel match lasts 90–120 min in two or three sets of 40 min [4], including 10–11 points lasting on average 13 to 15 s [5] with similar resting times (14–16 s) between points [6]. The game intensity can be determined by the rhythm of play (i.e., the ratio of actions and point duration, expressed as shots per s). Earlier studies found ratios of six to eight shots per point [6] resulting in 0.8 to 0.9 shots per s [7]. Interestingly, the intensity seems not to be affected by the set or the point duration [5,8]. Thus, taken together, these data yielded that a padel player would perform a volume of 945 to 1215 hits after a two-set match, and 1260 to 1620 after a three-set match. While interesting for training and coaching, these data are extracted from different studies and, thus, require confirmation.

High-level padel players use 10 to 40 technical actions, involving forehand and backhand directs, volleys, wall bounces and smashes [9,10]. Technical actions can be classified according to the area of the court where they are executed, considering groundstrokes and net strokes [11]. The use of particular technical actions may define the effectiveness of the game. For instance, the ability to play close to the net increases the chances of winning the point [12]. To maintain the net, players use volleys which have low error rates and a high likelihood of game continuity [13]. To recover the net, players can use lobs (to send back the opponent to the backcourt) or counterattack an opponent’s lobs by “bandeja” or smash [14,15]. Moreover, players can win the point from the backcourt by directs or wall-bounce hits (e.g., “bajada”), but the likelihood of committing an error increases [16]. While padel technical dynamics are well understood [9], the fluctuations in the use of particular technical actions during a match remain unexplored.

Given the high volumes of actions performed during a set, it can be anticipated that players may alter their game patterns as a result of the fatigue incurred during the game. In modern padel, around 40% of matches end up being three sets [17], resulting in greater accumulated fatigue [18,19,20]. Although the large resting times in padel can mitigate the onset of fatigue [21], the minimal appearance of physical fatigue during long points can reduce players’ reaction times and accuracy [22]. Thus, a better understanding of the players’ response to fatigue during the sets seems required to better prepare players according to the needs of the competition.

This study aimed to determine the distributions of number of shots per point, point duration, point pace and technical actions during the match in professional men and women padel players. Because the set and gender influence game parameters such as the number of breaks, winners and errors [23], we hypothesized that changes in the volume and the use of particular technical actions are a result of adapting the style of play to the incurred fatigue. In turn, no changes in the intensity are expected [5,8].

## 2. Materials and Methods

### 2.1. Experimental Approach to the Problem

Understanding players’ performance in different match situations would help coaches to improve periodization, training and match management. Therefore, the number of shots per point, point duration, point pace (shots per second) and technical actions (18 stroke types) during the game were assessed by systematic observation. Observational designs have been successfully used to measure padel performance [9,12] and allow for the objective assessment of emerging player responses in a natural competitive context. The data obtained were processed using an automated classification method, such as descriptive analysis, which provides an insightful explanation of the data and their usefulness in practical settings.

### 2.2. Sample

Twenty matches of the 2020 men’s and women’s World Padel Tour (WPT) were analyzed by systematic observation. Quarterfinals, semifinals and finals matches were included to ensure a highly competitive level. The final sample comprised 35,145 hits corresponding to 3239 points (men = 1602; women = 1637). The men players (*n* = 16; age = 26.89 ± 7.02 years; height = 178.26 ± 6.90 cm; laterality = 5 left-handed + 11 right-handed) and the women players (*n* = 16; age = 26.01 ± 6.67 years; height = 169.20 ± 6.21 cm; laterality = 4 left-handed + 12 right-handed) had professional experience competing in WPT tournaments. Matches with any number of sets and golden points were included. Matches were obtained from the official WPT website, from which they were downloaded for the observation and data recording process. Variables were collected through systematic observation by two sports analysts (observers) specialized in padel, trained for this task, using the specialized software LINCE v2.1.0. [24], and we designed an ad-hoc instrument to analyze the variables under study. At the end of the training process, each observer analyzed the same set to calculate the inter-observer reliability through Multirater Kappa Free [25], as presented in Table 1. The procedures were reviewed and approved by the Ethics Committee.

### 2.3. Procedures

In this descriptive study, data from professional padel competitions were retrieved from official video matches following sports observational analysis methods for a nomothetic, punctual and multidimensional design [26]. Number of shots per point, point duration and point pace, defined as the ratio between the number of shots per point and the point duration, expressed as the number of shots per second [4,27], were measured. Technical actions were classified into 18 types, distinguishing net and groundstroke actions, as described in Table 2 [28].

### 2.4. Statistical Analysis

Descriptive analysis included mean, standard deviation (SD), median, maximum, minimum, 25th and 75th percentiles (i.e., interquartile range, IQR) and 95% confidence intervals (CIs). Outliers were identified as values exceeding 1.5 times the IQR and excluded from the analysis. Because data followed a non-normal distribution (Kolmogorov–Smirnov tests *p* < 0.05) and were positively skewed, we conducted non-parametric tests to determine the differences in number of shots per point, point duration, point pace and technical actions between men and women (Mann–Whitney test) and sets (Kruskal–Wallis tests). Rank–Biserial correlation was used to estimate the effect size of Mann–Whitney test. Dunn’s post-hoc test with Bonferroni correction was performed to identify differences between sets. Rank eta squared (Eta2) was calculated to determine the effect size of the Kruskal–Wallis test, interpreted as small (0.01–0.06), moderate (0.06–0.14) and large (>0.14) [29]. The significance level was set at *p* < 0.05. Calculations were done in JASP v.0.19. Visualizations were done with Flourish studio.

## 3. Results

**Figure 1:**
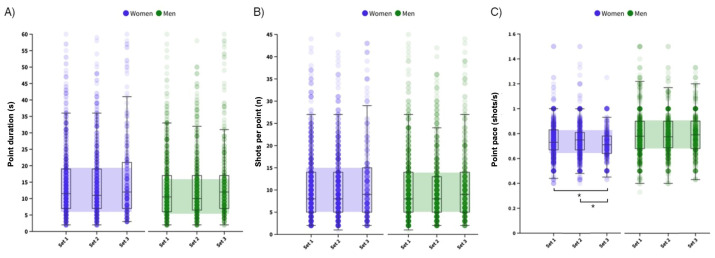
Distribution of point duration (**A**), number of shots per point (**B**) and point pace (**C**) between women (blue dots, right side of the panels) and men (green dots, left side of the panels). Boxes comprise the 25th and 75th percentiles. Medians are shown by the horizontal lines inside the boxes. Minimum and maximum values are shown as lines outside the boxes. * Significant differences between sets (*p* < 0.05).

**Figure 2:**
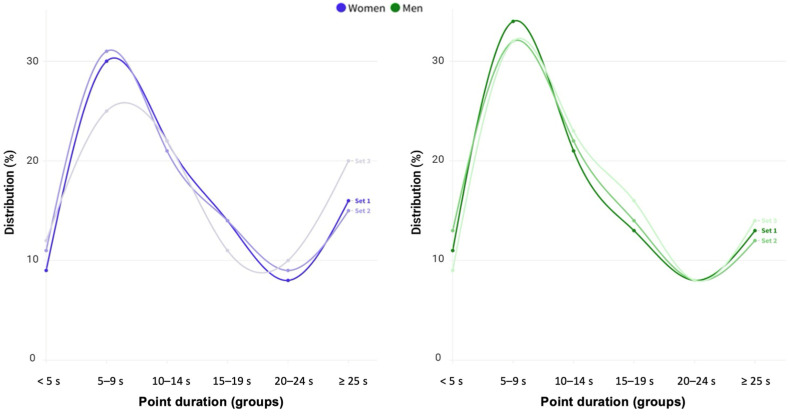
Distribution of point duration groups along the set in women (blue dots, **left panel**) and men (green dots **right panel**).

The final sample comprised 35,145 hits corresponding to 3239 points. Men’s points (Figure 1) lasted 13.5–14.8 s, comprising 10–11 hits, resulting in a ratio of 0.80 shots per second, with no differences between sets (point duration: *p* = 0.171, Eta2 < 0.001; number of shots: *p* = 0.124, Eta2 < 0.001; point pace *p* = 0.993, Eta2 < 0.001). Women’s points (Figure 1) lasted longer than men’s (14.4 to 16.2 s, *p* = 0.011, r = 0.052) but included a similar number of shots per point (*p* = 0.910, r = 0.002), resulting in a lower point pace (0.73–0.75 shots per second, *p* < 0.001, r = 0.200), particularly in the third set (*p* = 0.014, Eta2 = 0.004). The analysis of point duration groups (Figure 2) found a similar distribution during the match in men (*p* = 0.171) and women (*p* = 0.418), depicting an inverted S-shape with particular groups more prevalent than others.

Table 3 (women) and Table 4 (men) show the distribution of technical actions during the sets. Lobs were used in >76% of the points, with a frequency of two to three times per point. Volleys and directs were used in >60% of the points, with a frequency of one to three times per point. Bandejas were used in 50% (men) and 60% (women) of the points, with a frequency of one to two times per point. Backwalls and flat smashes were used at least once in 30–48% of the points. The remaining actions were used in less than 30% of the points. Selective actions had a greater frequency per point during the third set both in women (flat smash, smash x3) and men (forehand/backhand volleys, side-wall forehand).

## 4. Discussion

The main findings of the study indicated the following: (I) a similar number of shots per point and point duration between men and women professional padel players, (II) a lower point pace in women as a result of longer points, particularly in the third set, (III) half of the points included, at least, two to three lobs, one to three volleys and directs, one to two bandejas and, likely, one smash and (IV) selective technical actions (bandeja, forehand direct and volleys) had a greater frequency of use in the third set, suggesting occasional changes in the style of play as a response to particular competitive situations. These findings contribute to the existing little knowledge on the relationship between number of shots per point, point duration, point pace and the use of particular technical action during a professional padel game. 

The number of shots per point and point duration were consistent during a padel match, with expected point duration between 5 and 20 s (mostly 5 and 9 s) involving five to fifteen hits, resulting in ratios of ~0.70 to 0.80 shots per second. Particularly, we found women padel players to have longer point duration and lower point pace during the third set, which concurs with earlier studies [5,27,30]. Arguably, these differences can be explained by the higher use of lobs [14], as demonstrated by our results, and crossed trajectories [15], increasing the time lapse between hits and resulting in a lower rhythm of play and longer point duration. All in all, although statistically significant, these minor differences might not be sufficiently important to require specific considerations for training between men and women in terms of number of shots per point, point duration and point pace.

A new contribution of this study is the assessment of frequencies of technical actions per point. Our results found that ~76–86% of the points included at least two to three lobs, ~50–60% included at least one to three volleys and directs, one to two bandejas and, likely, one smash, and ~30–50% included at least one backwall and one flat smash. Accordingly, these big-six actions constitute the foundations of padel offensive and defensive game and must be acheived to reach a proficiency level. Lobs are considered the main option to achieve net positions [14]. Volleys are essential to both solving the point and maintaining an advantageous offensive position close to the net [3]. Similarly, at the baseline, directs are used to send the opponents back and recover the net position [14], and constitute a more conservative option to continue the point compared to backwall strokes, most likely to result in an error [13]. Bandejas are key defensive actions to maintain the net after a lob, continue the point in an advantageous position close to the net and avoid counterattacks [31]. Smashes are the primary final actions to solve the point [15,32], being more effective when performed near the net [33]. The remaining technical actions examined were used in less than 30% of the points; they are equally important but reserved for specific tactical conditions. For instance, the contrapared is a last-ditch action to reply to a backcourt ball when there is no time to adopt a well-orientated offensive position. In sum, this information is particularly valuable to design technical–tactical drills that simulate real competitive scenarios, in which players have limited options to hit the ball and solve the point.

In padel, the use of a particular action is strongly associated with the preceding one [9]. Thus, the observed increase in a given technical action may be explained by occasional changes in the style of play as a response to particular competitive situations. Another plausible explanation for these technical changes could be the fatigue incurred during the match. However, considering the sufficient recovery periods between points and the low physical demands in padel [21,34], it seems unlikely to explain changes in game style resulting from the potential onset of fatigue. Future studies should aim to clarify the reasons for the variation in the use of each particular action. However, the results suggest that fatigue has minimal impact on playing style, as players are required to execute specific strokes consistently throughout each set.

This work explained how game volumes, intensity and technical actions fluctuate during professional padel matches in men and women players. Data provide new insights on how technical actions are expected to occur during a point. This information may serve to establish benchmarks during practice, identify players’ ability to respond to professional physical and technical demands and, ultimately, design tailored training sessions with boundaries to solve the point simulating common match situations. For example, coaches can use, in training sessions, drills lasting ~15 s, comprising 10–11 hits including two to three lobs, one to three volleys and directs, one to two bandejas and one smash, to stimulate the demands of match play, creating a suitable adaptation that can be extrapolated to competition. This factor can also be extrapolated to coaches and researchers of other racket sports, where they include and analyze the minimum parameters of the game in relation to the duration of the point, minimum number of strokes and specific minimum strokes. They must also be able to work on the basis of these parameters, for example, in tennis, to establish training exercises that last for a certain duration, where a certain number of forehands or slices, and parallels or crosses are included as a minimum. On the other hand, it is possible to adjust the premises of the exercises in training based on what happens in competition, with the coach adapting the premises based on the offensive or defensive nature of the pair. Accordingly, specific offensive drills should allow players to solve the point using no more than three volleys, while defensive drills should improve players’ ability to recover the net using no more than two lobs and two bandejas. These boundaries are consistent with young padel players [6] and, thus, may represent a benchmark for padel practice at formative levels.

With regard to point pace, it seems to be a determinant in reaching the professional level, with players having less than 1 s to hit the ball. It is recommended that coaches integrate into training sessions, either as part of the warm-up or as a primary component, scenarios that facilitate a high-paced hitting rhythm. This may be achieved by reducing the available space on the court or initiating the point with a hitting sequence that requires two strokes to be executed within a brief timeframe.

Furthermore, a better knowledge of the variation in the use of particular actions during the set allows for targeted training and development of specific sequences and tactical situations. According to our findings, bandejas, volleys and directs are determinants during the third set of the match, thus requiring special attention during the final stages of the match. It is recommended that coaches consider increasing the number of these strokes in the final part of the session, depending on the technical nature of the session. For example, limiting the number of lobs could be an effective method to increase the number of volleys, while limiting the number of wall strokes could be an effective method to increase the use of direct strokes.

In sum, a comprehensive analysis of point duration, pace of play and number of actions provides essential information for the development of training sessions that simulate the demands of competition. The data presented here can assist coaches of padel in designing specific exercises that allow for a minimum and maximum number of strokes of a certain type, thereby reinforcing the volume of play, intensity and number of strokes in accordance with the demands of professional competition. Furthermore, it can assist coaches and researchers of other racket sports in adapting these results based on the characteristics of their respective sports.

Nevertheless, this study has some limitations that should be considered to better interpret the findings. The lack of confounding variables may alter the current findings, mainly the scoreboard, the players’ streak (e.g., hot-hand and momentum) and the players’ characteristics (side of play, height, ranking position and technical ability). Conversely, despite the reliability of the methodology employed for data collection via video observation, the potential for observer bias remains, even with the implementation of reliability checks. To ensure the validity of the tool for future research, it is essential to validate it further through the analysis of the variables in question. Future studies should address these limitations to determine the game parameters of professional padel players more accurately. Furthermore, experimental studies are now required to demonstrate the effectiveness of specific technical, tactical and physical training interventions in padel players’ performance based on the current findings.

## 5. Conclusions

In conclusions, professional men and women padel players display a similar and consistent game volume, intensity and technical actions during the match, with minimal variations along the sets. These findings indicate that fatigue during the match has a minimal impact on the style of play and remains consistent across all three sets. Technically, the game is mostly defined by six big actions, with the following expected occurrence: two to three lobs in ~76–86% of the points, one to three volleys and directs, one to two bandejas and one smash in ~50–60% of the points and one backwall and one flat smash in ~30–50% of the points. Selective technical actions (bandeja, forehand direct and volleys) had a a greater frequency of use in the third set, suggesting occasional changes in the style of play as a response to particular competitive situations. The lack of practical meaningful differences seems to indicate the relevance of tactical decisions over physical or technical domains at professional padel level.

## Figures and Tables

**Table 1:** Inter-observer and intra-observer reliability.

	Intra-Observer	Inter-Observer
Study Variables	*K*	
Sex	1.00	1.00
Set	1.00	1.00
Number of shots per point	1.00	0.95
Point duration	0.82	0.79
Point pace	1.00	0.98
Technical actions	1.00	0.94
Total	0.97	0.94

**Table 2:** Padel technical action classification.

**Net Actions**	**Description**
Volley	Stroke without a bounce that is hit with a short up and down motion of the padel. Subtypes: forehand volley and backhand volley.
Bandeja	Stroke hit without bouncing that is hit from the player’s dominant side, usually further away from the net. It is considered an intermediate stroke between the smash and the forehand volley, as the point of impact is lower than the powerful smash and higher than the volley.
Topspin bandeja	Variant of the bandeja that is executed with a topspin effect.
Smash	Stroke without a bounce, taken over the player’s head, with an up and down motion of the padel racket. A distinction is made between flat smash (smash with a flat spin) and X3 smash (smash in which the ball, once hit and after the bounce on the ground, leaves the court over the fences with a height of 3 m).
Feint smash	The player executes the stroke in the same position as the smash, but in the impact phase opts for a precise, placed stroke, rather than the more powerful stroke of a smash.
**Background Actions**	**Description**
Direct	Hit at medium height after the ball bounces in the court. Subtypes: direct forehand and direct backhand.
Side wall	The ball, after bouncing on the ground, bounces off the side wall. Subtypes: side-wall forehand and side-wall backhand.
Backwall	The ball, after bouncing on the ground, bounces off the backwall glass. Subtypes: backwall forehand and backwall backhand.
Double wall	The ball, after bouncing on the ground, bounces off two walls. Subtypes: open double wall (after bouncing on the ground, first touches the side wall and then the back wall) and close double wall (after bouncing on the ground, first touches the back wall and then the side wall).
Contrapared	Hit the ball to the glass, either the back or side, to pass to the opponent’s court.
Bajada	Stroke played at the back of the court, of an offensive nature, resulting from a hit after the ball has struck the back glass at a height above or equal to the shoulders.
Lob	The technique involves sending the ball in an upward trajectory to pass over opposing players, to overtake them and force them back into defensive positions.

Forehand: stroke from the player’s dominant side; Backhand: stroke from the player’s non-dominant side.

**Table 3:** Differences in the use of technical actions during the sets in professional women padel players.

Women	Set 1			Set 2			Set 3			*p*	Eta2
	*n* Use	% Use	95% CI	*n* Use	% Use	95% CI	*n* Use	% Use	95% CI		
**Net Actions**											
Forehand volley	424	60%	2.0–2.2	383	59%	1.9–2.2	157	57%	2.0–2.4	0.521	<0.001
Backhand volley	450	63%	2.1–2.4	397	61%	1.9–2.2	163	59%	2.0–2.5	0.274	<0.001
Bandeja	426	60%	2.1–2.5	428	66%	2.1–2.4	169	61%	2.4–2.9	0.827	<0.001
Topspin bandeja	147	21%	1.3–1.5	125	19%	1.1–1.4	44	16%	1.0–1.4	0.193	<0.001
Flat smash	159	22% ^c^	1.0–1.1	131	20% ^c^	1.0–1.1	86	31% ^a,b^	1.0–1.1	0.001 *	0.007
Smash x3	31	4% ^c^	1.0–1.1	32	5% ^c^	1.0–1.0	24	9% ^a,b^	1.0–1.0	0.021 *	0.004
**Background Actions**											
Direct Forehand	468	66%	2.0–2.3	423	65%	1.9–2.1	190	69%	2.2–2.6	0.095	0.002
Direct Backhand	531	75%	1.9–2.2	489	75%	1.9–2.1	201	73%	1.8–2.2	0.651	<0.001
Side-wall Forehand	49	7%	1.0–1.1	66	10%	1.0–1.1	23	8%	1.0–1.2	0.101	0.002
Side-wall Backhand	134	19%	1.0–1.1	112	17%	1.0–1.1	58	21%	1.0–1.1	0.386	<0.001
Backwall Forehand	297	42%	1.6–1.9	272	42%	1.5–1.7	132	48%	1.6–1.8	0.273	<0.001
Backwall Backhand	220	31%	1.3–1.5	192	29%	1.3–1.5	90	33%	1.2–1.5	0.676	<0.001
Double wall open	165	23%	1.1–1.2	137	21%	1.1–1.3	63	23%	1.1–1.4	0.618	<0.001
Double wall close	109	15%	1.1–1.3	111	17%	1.1–1.2	48	17%	1.1–1.4	0.596	<0.001
Contrapared	52	7%	1.1–1.3	52	8%	1.0–1.2	20	7%	0.9–1.1	0.880	<0.001
Bajada	162	23%	1.2–1.3	141	22%	1.1–1.3	68	25%	1.0–1.3	0.387	<0.001
Lobs	615	86%	3.0–3.4	564	86%	2.8–3.2	235	85%	3.2–3.9	0.519	<0.001

Note. *n*: frequency; % use: indicates the percentage of points including a given type of stroke; 95% CI: confidence interval; * bold letter: *p* < 0.05; a, b and c: post-hoc Bonferroni *p* < 0.05, Set 1a, Set 2b, Set 3c; Eta2: eta squared effect size.

**Table 4:** Differences in the use of technical actions during the sets in professional men padel players.

Men	Set 1			Set 2				Set 3		*p*	Eta2
	*n* Use	% Use	95% IC	*n* Use	% Use	95% IC	*n* Use	% Use	95% IC		
**Net Actions**											
Forehand volley	430	64%	1.9–2.2	339	58% ^c^	1.9–2.0	228	64% ^b^	2.2–2.6	0.042 *	0.003
Backhand volley	472	71% ^c^	2.2–2.6	427	74% ^c^	2.3–2.7	277	78% ^a,b^	2.6–3.0	0.002 *	0.006
Bandeja	295	44%	2.0–2.3	276	48%	1.7–2.1	165	46%	2.1–2.6	0.677	<0.001
Topspin bandeja	139	21%	1.2–1.3	117	20%	1.2–1.4	82	23%	1.2–1.4	0.571	<0.001
Flat smash	202	30%	1.0–1.1	177	31%	1.0–1.1	118	33%	1.0–1.1	0.572	<0.001
Smash x3	51	8%	1.0–1.2	45	8%	1.0–1.1	26	7%	0.9–1.1	0.967	<0.001
Feint smash	30	4%	1.0–1.2	34	6%	0.9–1.2	15	4%	0.9–1.1	0.943	<0.001
**Background Actions**											
Direct Forehand	428	64%	1.6–1.8	347	60%	1.7–1.9	220	62%	1.7–2.0	0.372	<0.001
Direct Backhand	445	67%	1.7–1.9	389	67%	1.7–1.9	251	71%	1.7–1.9	0.350	<0.001
Side-wall Forehand	49	7% ^b^	1.0–1.1	67	12% ^a^	1.0–1.1	30	8%	1.0–1.2	0.034 *	0.003
Side-wall Backhand	151	23%	1.1–1.1	151	26%	1.1–1.1	85	24%	1.1–1.2	0.433	<0.001
Back wall Forehand	285	43%	1.5–1.7	241	42%	1.5–1.8	160	45%	1.5–1.9	0.414	<0.001
Back wall Backhand	237	36%	1.3–1.5	196	34%	1.2–1.4	129	36%	1.4–1.7	0.473	<0.001
Double wall open	109	16%	1.1–1.3	104	18%	1.1–1.2	62	17%	1.1–1.3	0.483	<0.001
Double wall close	80	12%	1.1–1.4	66	11%	1.0–1.3	49	14%	1.1–1.4	0.499	<0.001
Contrapared	74	11%	1.0–1.2	75	13%	1.1–1.2	44	12%	1.0–1.2	0.579	<0.001
Bajada	109	16%	1.1–1.3	101	17%	1.1–1.3	50	14%	1.2–1.3	0.357	<0.001
Lobs	510	76%	2.4–2.8	448	77%	2.4–2.8	288	81%	2.5–3.0	0.425	<0.001

Note. *n*: frequency; % use: indicates the percentage of points including a given type of stroke; 95% CI: confidence interval; * bold letter: *p* < 0.05; a, b and c: post hoc Bonferroni *p* < 0.05, Set 1a, Set 2b, Set 3c; Eta2: eta squared effect size.

## Data Availability

The original contributions presented in the study are included in the article, further inquiries can be directed to the corresponding author.
